# Age‑dependent and post‑intraventricular hemorrhage remodeling of the ependymal glycocalyx in mice

**DOI:** 10.1186/s12987-025-00725-x

**Published:** 2025-11-07

**Authors:** Tomohiro Iida, Kosuke Mori, Hiroyuki Tomita, Kazufumi Ohmura, Kohtaro Taguchi, Ayumi Niwa, Tomohiro Kanayama, Shigeyuki Sugie, Hideshi Okada, Tsuyoshi Izumo, Akira Hara

**Affiliations:** 1 Department of Neurosurgery, Gifu University Graduate School of Medicine, 1-1 Yanagido, Gifu, 501-1194 Japan; 2 Department of Tumor Pathology, Gifu University Graduate School of Medicine, 1-1 Yanagido, Gifu, 501-1194 Japan; 3 Center for One Medicine Innovative Translational Research, Gifu University Institute for Advanced Study, Gifu, Japan; 4 Department of Pathology, Asahi University Hospital, Gifu, Japan; 5 Department of Emergency and Disaster Medicine, Gifu University Graduate School of Medicine, 1-1 Yanagido, Gifu, 501-1194 Japan

**Keywords:** Ependymal glycocalyx, Brain–cerebrospinal fluid barrier, Lectin, Electron microscopy, Aging, Intraventricular hemorrhage

## Abstract

**Background:**

The ependymal glycocalyx (Gcx) is a glycan-rich apical structure that lines the ventricular brain surface. It is thought to contribute to cerebrospinal fluid dynamics and brain homeostasis by forming a selective barrier, preserving surface charge, and supporting ciliary function. Despite its importance, the structural integrity and glycan composition of the ependymal Gcx remain poorly understood, particularly in the context of physiological aging and acute neurological injury, such as intraventricular hemorrhage (IVH). We aimed to elucidate the physiological role of the ependymal Gcx and its alterations in response to aging and acute brain injury.

**Methods:**

We comprehensively investigated age- and injury-related changes in the ependymal Gcx using young (8–10-week-old), aged (60–62-week-old), and IVH model mice. The Gcx structure was visualized using lanthanum-enhanced electron microscopy, and glycan profiles were assessed through double immunofluorescence staining with S100β and a panel of 21 fluorescent lectins. Gcx thickness was quantitatively analyzed using a novel image analysis approach based on fluorescence intensity profiles. Single-cell RNA sequencing (scRNA-seq) was performed on ventricular tissue to identify transcriptional changes in aged ependymal cells related to glycan biosynthesis, glycan sialylation, desialylation, vesicular transport, and inflammatory responses.

**Results:**

Immunohistochemistry showed that, in young mice, the ependymal Gcx was bound to SiaFind and Lycopersicon esculentum lectin; additionally, it is bound to PNA even without desialylation. In aged mice, the Gcx displayed marked thinning, detachment, and significant loss of terminal sialic acids. In young mice, Gcx disruption after IVH peaked on day 3 and correlated with periventricular inflammation; in contrast, the inflammation persisted in aged IVH mice. Integrated single-cell RNA-seq revealed age-related alterations. Key sialylation genes (ST3GAL1, SLC35A1) and core 1 O-glycan enzymes (C1GALT1, C1GALT1C1) were downregulated, whereas ST3GAL5 and plasma-membrane sialidase NEU3 were upregulated. Additionally, senescence markers (Cdkn1a, Trp53) and multiple interferon-stimulated genes were elevated.

**Conclusions:**

The ependymal Gcx is a dynamic and injury-sensitive structure whose integrity is compromised by aging and IVH. Its disruption promotes neuroinflammation and may contribute to the development of hydrocephalus and neurodegeneration. Therapeutic modulation of glycosylation pathways may provide a promising strategy to preserve Gcx function and protect the internal brain environment.

**Supplementary Information:**

The online version contains supplementary material available at 10.1186/s12987-025-00725-x.

## Background

The ependymal cells lining the cerebral ventricles are ciliated glial cells derived from radial glia [1], and they play a pivotal role in cerebrospinal fluid (CSF) circulation, molecular exchange with the brain parenchyma, and maintenance of the neural stem cell niche [2]. These cells are covered by the glycocalyx (Gcx), a complex of polysaccharides and proteins [3–6], which functions as the frontline interface mediating cellular interactions with the environment. The ependymal Gcx may contribute to the formation of a negatively charged cell surface, support CSF circulation, and act as a physical and immunological barrier against pathogens and metabolic waste [3, 7].

However, the structural and compositional alterations of the ependymal Gcx in response to physiological and pathological stressors such as aging or acute brain injury and the subsequent effect on ependymal cell function and overall brain homeostasis remain unclear. Few studies have clearly distinguished between surface Gcx and intracellular glycans.

Therefore, in the present study, we conducted a comprehensive histochemical analysis using a panel of lectins with diverse glycan-binding specificities to qualitatively and quantitatively assess changes in the structure, composition, and localization of the lateral ventricular ependymal Gcx in young adult mice, aged mice, and mice with intraventricular hemorrhage (IVH). In addition, we evaluated Galectin-3, a lectin upregulated in glial cells under inflammatory conditions and constitutively expressed in ependymal cells, as a potential indicator of ventricular inflammation [8, 9], as well as Kolmer cells, which are macrophage-like cells residing on the choroid plexus surface that participate in immune surveillance of the CSF [10]. Through this approach, we aimed to elucidate not only the physiological role of the ependymal Gcx but also its alterations in response to aging and acute brain injury, thereby providing insights that may contribute to the development of diagnostic markers and therapeutic strategies.

## Methods

### Mice

Wild-type C57BL/6J mice were obtained from the Jackson Laboratory Japan, Inc. (Yokohama, Japan). Male mice aged 8–10 and 60–62 weeks were used in all experiments. In this study, 8-10-week-old mice were defined as “young adult” and 60–62-week-old mice were defined as “aged”. All animal procedures were conducted in accordance with the guidelines of the Gifu University International Animal Care and Use Committee (Approval No. A20250001). Mice were housed under standard laboratory conditions with a 12 h light/12 h dark cycle at a controlled temperature of 22 °C, with ad libitum access to food and water.

### IVH animal model

The IVH mouse model was established via intracerebroventricular (i.c.v.) injection of 25 µL of autologous whole blood, as previously described [11]. Mice were anesthetized by intraperitoneal administration of a combination of medetomidine (0.3 mg/kg), midazolam (4.0 mg/kg), and butorphanol (5.0 mg/kg), as reported previously [12]. A 1-mm burr hole was drilled in the skull at a point 0.5 mm posterior and 1.0 mm lateral to the bregma. A 26-gauge needle was inserted freehand into the right lateral ventricle to a depth of 2.5 mm. A total of 25 µl of autologous whole blood, collected from the orbital venous plexus without anticoagulants, was immediately injected—within seconds of collection—over approximately 10 s using a Hamilton microsyringe. After injection, the needle was left in place for an additional 2 min to minimize backflow. The burr hole was sealed with bone wax, and the skin incision was closed with sutures.

### Tissue preparation of mice

Frozen sections: Mice were euthanized, and tissues were collected without perfusion. Samples were embedded in an optimal cutting temperature compound, snap-frozen in liquid nitrogen, and stored at -80 °C. The embedded tissues were coronally sectioned at a thickness of 5 μm near the optic chiasm using a rotary microtome (Leica, Wetzlar, Germany).

Paraffin sections: Following anesthesia, the thoracic cavities of the mice were opened, and the inferior vena cava was incised. Perfusion was performed using a drip infusion system, first with cold phosphate-buffered saline (PBS; 25 ml at approximately 3 ml/min), followed by an equal volume of cold 4% paraformaldehyde (PFA; 25 ml at approximately 3 ml/min). Afterward, the tissues were harvested, dissected into smaller pieces, and processed for paraffin embedding and sectioning.

### Lectin fluorescent staining

Three lectin kits (I–III), each comprising a diverse array of lectins with distinct binding specificities for broad screening of glycan structures on cell surfaces, tissues, or purified glycoproteins, were obtained from Vector Laboratories (Burlingame, CA, USA). In addition, SiaFind α2,3-Specific Lectenz, an antibody-based reagent that specifically recognizes α2,3-linked sialic acid residues, was purchased from Lectenz Bio (Athens, GA, USA). Details of the biotinylated lectins included in kits I–III and SiaFind are summarized in Table 1.

For double-fluorescence staining using an antibody and biotinylated lectins, tissue sections were fixed in 4% PFA in PBS for 15 min. After washing with PBS, sections were blocked with the Histofine Mouse Stain Kit (NICHIREI BIOSCIENCES INC.) for 60 min at room temperature. Subsequently, biotinylated lectins (1:200 dilution) and an ependymal cell marker, the S100β antibody (mouse monoclonal, 1:500 dilution; sc-393919, Santa Cruz Biotechnology), were applied and incubated overnight at 4 °C. The following day, sections were washed with PBS, blocked again with the Histofine Mouse Stain Kit, and incubated with Alexa Fluor 488-conjugated anti-mouse secondary antibody (1:250 dilution; ab150165, Abcam) and DyLight 594-conjugated streptavidin (1:250 dilution; Vector Laboratories) for 1 h at room temperature. Finally, after washing with PBS, the nuclei were counterstained with DAPI, and coverslips were mounted using appropriate mounting medium. Ependymal cells were identified based on their S100β-positivity and their characteristic localization as a single layer lining the ventricular wall.

**Table 1:** Binding specificities of the lectins that were used in this study

Lectin	Common Abbreviation	Specificity
Concanavalin A	ConA	αMan > αGlc
Dolichos biflorus agglutinin	DBA	GalNAcα(1,3)
Peanut agglutinin	PNA	Galβ(1,3) > Galβ(1,4) > Gal
Ricinus communis agglutinin Ⅰ	RCA Ⅰ	GalNAc > αGal
Soybean agglutinin	SBA	αGalNAc > αGal > βGalNAc
Ulex Europaeus agglutinin Ⅰ	UEA Ⅰ	Fucα(1,2)
Wheat Germ agglutinin	WGA	NeuAc >>>GlcNAc
Griffonia simplicifolia lectin Ⅰ	GSL Ⅰ	αGal
Lens culinaris lectin	LCA	αMan > αGlc
Phaseolus vulgaris Erythroagglutinin	PHA E	Complex structures
Phaseolus vulgaris Leucoagglutinin	PHA L	Complex structures
Pisum sativum agglutinin	PSA	αMan > αGlc
Wheat Germ agglutinin, succinylated	succinylated WGA	GlcNAc
Datura stramonium lectin	DSL	GlcNAc oligomer
Erythrina cristagalli lectin	ECL	Gal
Griffonia simplicifolia lectin Ⅱ	GSL Ⅱ	GlcNAc
Jacalin	Jacalin	αGal
Lycopersicon esculentum lectin	LEL	GlcNAc oligomer
Solanum tuberosum lectin	STL	GlcNAc
Vicia villosa lectin	VVL	GalNAc
SiaFind α2,3-Specific Lectenz	SiaFind	α2,3 linked NeuAc

Abbreviation: Man, mannose; Glc, glucose; GalNAc, N-acetylgalactosamine; Gal, galactose; Fuc, fucose; NeuAc, sialic acid; GlcNAc, N-acetylglucosamine

### Immunofluorescence staining

The paraffin-embedded brain tissues were cut into 4-µm sections. The sections were blocked with bovine serum albumin at 37 °C for 60 min; thereafter, they were incubated with the following primary antibodies overnight at 4 °C: ionized calcium binding adaptor molecule-1 (Iba-1) (1:500, #019-1974, Wako) and Galectin-3 (1:100, #13-5301-85, eBiosciences). After washing the sections three times with PBS, they were incubated with secondary antibodies at 37 °C for 60 min.

### Scanning electron microscopy (SEM)

Ependymal Gcx was visualized using SEM, as previously described [13, 14]. Briefly, tissue samples were cut into 5 mm³ sections and initially fixed for 2 h in a solution of 2% glutaraldehyde, 2% sucrose, 0.1 M sodium cacodylate buffer (pH 7.3), and 2% lanthanum nitrate. Thereafter, the specimens were immersed overnight in a solution containing 2% sucrose, 0.1 M sodium cacodylate buffer (pH 7.3), and 2% lanthanum nitrate, followed by washing in an alkaline solution (0.03 M sodium hydroxide with 2% sucrose). After fixation and washing, the specimens were dehydrated through a graded ethanol series. Subsequently, they were frozen in 100% ethanol and rapidly cooled with liquid nitrogen to create fracture surfaces for SEM observation. Afterward, the frozen tissues were fractured using a carving knife. Finally, the specimens were examined using SEM (S-4800, Hitachi, Tokyo, Japan).

### Low-vacuum scanning electron microscopy (LVSEM)

Regarding LVSEM, we used a modified version of a previously described method [15], the details of which are described in a manuscript in preparation at the time of the present study (Mori, K. et al.). Briefly, mice were deeply anesthetized and perfused with a solution of 10% neutral buffered formalin containing 1% Alcian Blue 8GX and 2% sucrose. Subsequently, the brains were harvested, processed for paraffin embedding, and cut into 2-µm thick serial sections. After deparaffinization, the sections were stained with Periodic Acid-Methenamine silver. The stained specimens were air-dried, mounted onto a sample holder using conductive adhesive tape, and observed without metal coating using a low-vacuum scanning electron microscope (TM3030Plus, Hitachi High-Tech, Tokyo, Japan) at an acceleration voltage of 15 kV.

### Transmission electron microscopy (TEM)

Tissue fixation was performed as described for the SEM. After fixation, brain samples were postfixed with 2% osmium tetroxide in 0.1 M cacodylate buffer for 2 h at 4 °C. Thereafter, the samples were dehydrated through a graded ethanol series (50–100%), treated with propylene oxide, and embedded in epoxy resin (Quetol812: DDSA: MNA = 7:4:4, with 1.5% DMP-30 catalyst). Polymerization was performed at 40 °C for 8 h, 70 °C for 24 h, and 75 °C for 12 h. Ultrathin Sect. (90 nm) were cut using an ultramicrotome (Leica EM UC7) and mounted on copper grids. Sections were stained with 2% uranyl acetate for 15 min, followed by lead citrate for 5 min, and dried on filter paper. Images were acquired using a transmission electron microscope (HT7800, Hitachi, Japan).

### Image analysis

Images used for fluorescence intensity and inter-inflection distance measurements were acquired under consistent imaging conditions to ensure data comparability. Following background subtraction, the mean fluorescence intensities of the Gcx and cytoplasm were measured in 10 randomly selected cells per animal (*n* = 3; total of 30 cells). The thickness of the ependymal Gcx was quantified as previously described [16], with minor modifications. Briefly, a measurement line was drawn perpendicular to the ependymal cell surface using merged images of lectin and S100β staining. Radial fluorescence intensity profiles for both channels were extracted at the ependymal surface and fitted with an 8th-degree polynomial function using ImageJ. This polynomial fit was chosen because it provides a smooth and flexible representation of fluorescence profiles, allowing direct visualization and precise determination of inflection points within the same environment used for image preprocessing. Inflection points were defined at the visually apparent transition point in the rising phase of the curve: the lectin-derived inflection point represented the outer boundary of the Gcx layer, and the S100β-derived inflection point represented the apical surface of the ependymal cell. The distance between these two inflection points was interpreted as the thickness of the ependymal Gcx. Gcx thickness was measured at 16 randomly selected points per animal (*n* = 3 or 5; total of 48 or 80 points). For validation, representative profiles were analyzed via sigmoidal (four-parameter logistic) fitting in GraphPad Prism, and the positions of the inflection points showed no significant differences from those obtained with the ImageJ-based method (Additional file 1). The extent of periventricular inflammation was evaluated by measuring Iba-1 fluorescence intensity within a 17-µm-wide band region of the periventricular brain parenchyma, as well as Galectin-3 fluorescence intensity in the ependymal cells. In addition, the size of Iba-1-positive Kolmer cells in the choroid plexus was assessed. The ventricular surface coverage of the Gcx was calculated from lectin-stained images as the proportion of the ependymal surface length that was positively stained for Gcx relative to the analyzable ventricular lining per section. To avoid confounding by ependymal loss, areas of denudation were excluded from quantitative analysis. Denudation was defined as S100β-negative gaps exposing the subependymal layer. This approach ensured that the reported percentages reflected Gcx changes only in morphologically intact ependyma.

### Data acquisition and analysis

We performed an integrated analysis of public single-cell RNA-sequencing (scRNA-seq) data comprising 9,406 murine ependymal cells. The dataset was compiled from five independent studies (accession IDs: SCP565, SRP135960, GSE74672, SCP318, and PMID_32669714_FACS) using the Talk2Data platform (v4, BioTuring Inc.).

### Bioinformatic analysis and visualization

All bioinformatic analyses, including cell type identification, segregation of young and aged adult populations, and differential gene expression analysis, were conducted within the Talk2Data environment. Per-cell normalization and log-transformation were applied using the platform defaults, and integration was performed within the same environment to handle cross-dataset effects. Library size distributions were inspected across age groups prior to differential testing. All figures were generated using the visualization tools integrated within the platform.

### Statistical analysis

All data are presented as mean ± standard error of the mean (S.E.M). Differences in Gcx thickness, periventricular coverage, Iba-1 fluorescence intensity per pixel, Kolmer cell Iba-1-positive area, and Galectin-3 fluorescence intensity between young and aged mice for each lectin were assessed using unpaired t-tests. Temporal changes in Gcx thickness, Iba-1 fluorescence intensity per pixel, Kolmer cell Iba-1-positive area, and Galectin-3 fluorescence intensity after IVH were analyzed via one-way analysis of variance (ANOVA) followed by Dunnett’s multiple-comparison test. To validate the use of inflection point distances as a quantitative measure of Gcx thickness, correlations with direct measurements were evaluated using Pearson’s correlation coefficient (r). *p* value < 0.05 was considered statistically significant. All statistical analyses were performed using GraphPad Prism 10.4.2 (GraphPad Software, Inc., La Jolla, CA, USA).

## Results

### Visualization and glycan profiling of the ependymal Gcx in normal mice

**Fig. 1:**
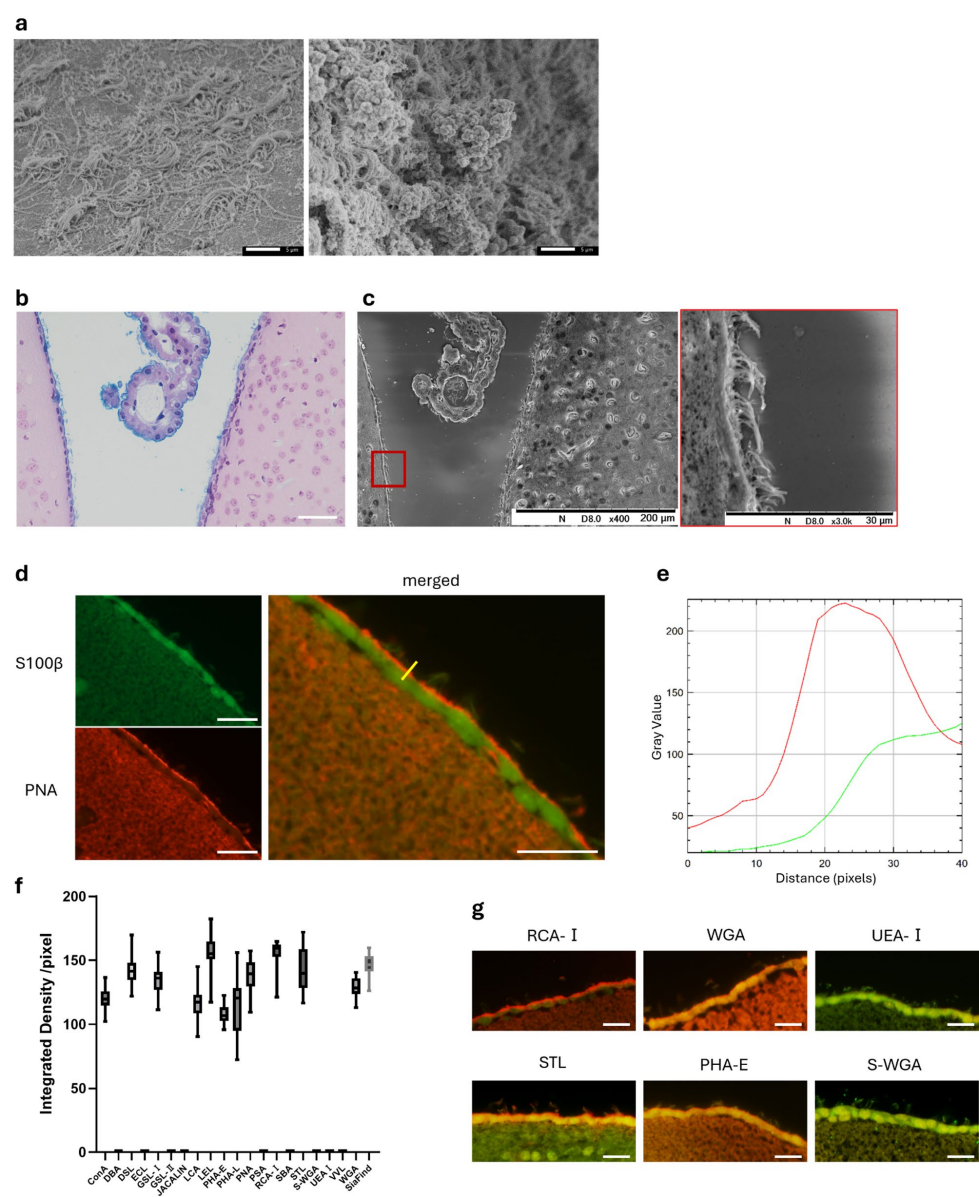
Visualization and glycan profiling of the ependymal Gcx in young adult mice. (**a**) Scanning electron microscopy (SEM) images of ventricular ependymal cells. Gcx is not visible under conventional glutaraldehyde fixation (left) but clearly visualized surrounding the cell surface and cilia with lanthanum staining (right). (**b**) Alcian blue + hematoxylin-eosin (HE) staining. Glycans covering the apical surface of the ependyma are stained blue with Alcian blue. White scale bar: 20 μm. (**c**) Low-vacuum SEM image of a serial section corresponding to (**b**). A three-dimensional view of the Gcx layer covering the base of the cilia and surrounding the ciliary shafts at the apical surface. Enlarged view on the right. (**d**) Double immunofluorescence staining of lectin (PNA, red) and S100β (green) in frozen sections. A distinct glycan layer is observed at the apical surface of the ependyma, comparable to that seen in electron microscopy. White scale bar: 10 μm. (**e**) Fluorescence intensity plot along the measurement line (yellow) in (**d**), from the ventricular lumen (left) toward the brain parenchyma (right); red indicates lectin, green indicates S100β. The plot confirms localization of the Gcx at the ependymal cell surface. (**f**) Bar graph showing the mean fluorescence intensity of the ependymal Gcx for each of the 21 lectins examined (*n* = 3 mice; total of 30 cells). (**g**) Merged images of lectin (red) and S100β (green) staining. Representative examples are shown: strong positive (RCA-I, STL), moderate positive (WGA, PHA-E), and negative (UEA-I, S-WGA). White scale bar: 10 μm

In SEM observations, the Gcx on the surface of ependymal cells was not detectable under conventional glutaraldehyde fixation. However, when combined with lanthanum staining, a dense Gcx layer was clearly visualized on the apical surface of ependymal cells and surrounding the cilia (Fig. 1a). Histologically, Alcian blue staining distinctly labeled the glycan layer on the apical membrane, and low-vacuum SEM enabled three-dimensional visualization of the Gcx structure covering the base of the cilia (Fig. 1b and c). In lectin staining, ependymal cells were immunolabeled with S100β, and the apical Gcx layer was clearly highlighted (Fig. 1d).

Furthermore, fluorescence-based screening using 21 lectins with diverse glycan-binding specificities revealed that the ependymal Gcx comprises a wide variety of glycans. Particularly, strong signals were observed for Lycopersicon esculentum lectin (LEL), Ricinus communis agglutinin I (RCA-I), α2,3-linked sialic acid-specific lectins (SiaFind), Solanum tuberosum lectin (STL), Datura stramonium lectin (DSL), and peanut agglutinin (PNA) [Figure 1f]. Representative images ranging from strongly positive to negative lectin signals are shown in Fig. 1g. In contrast, cytoplasmic binding was prominent for Vicia villosa lectin, Griffonia simplicifolia lectin I, Erythrina cristagalli lectin, Wheat germ agglutinin (WGA), LEL, and Succinylated Wheat Germ agglutinin (S-WGA). Notably, lectins such as PNA, which showed strong affinity to the apical Gcx, were negative in the cytoplasm (Fig. 1d), indicating distinct glycan profiles between the apical surface and intracellular compartments.

### Structural and compositional changes in the ependymal Gcx associated with aging

**Fig. 2:**
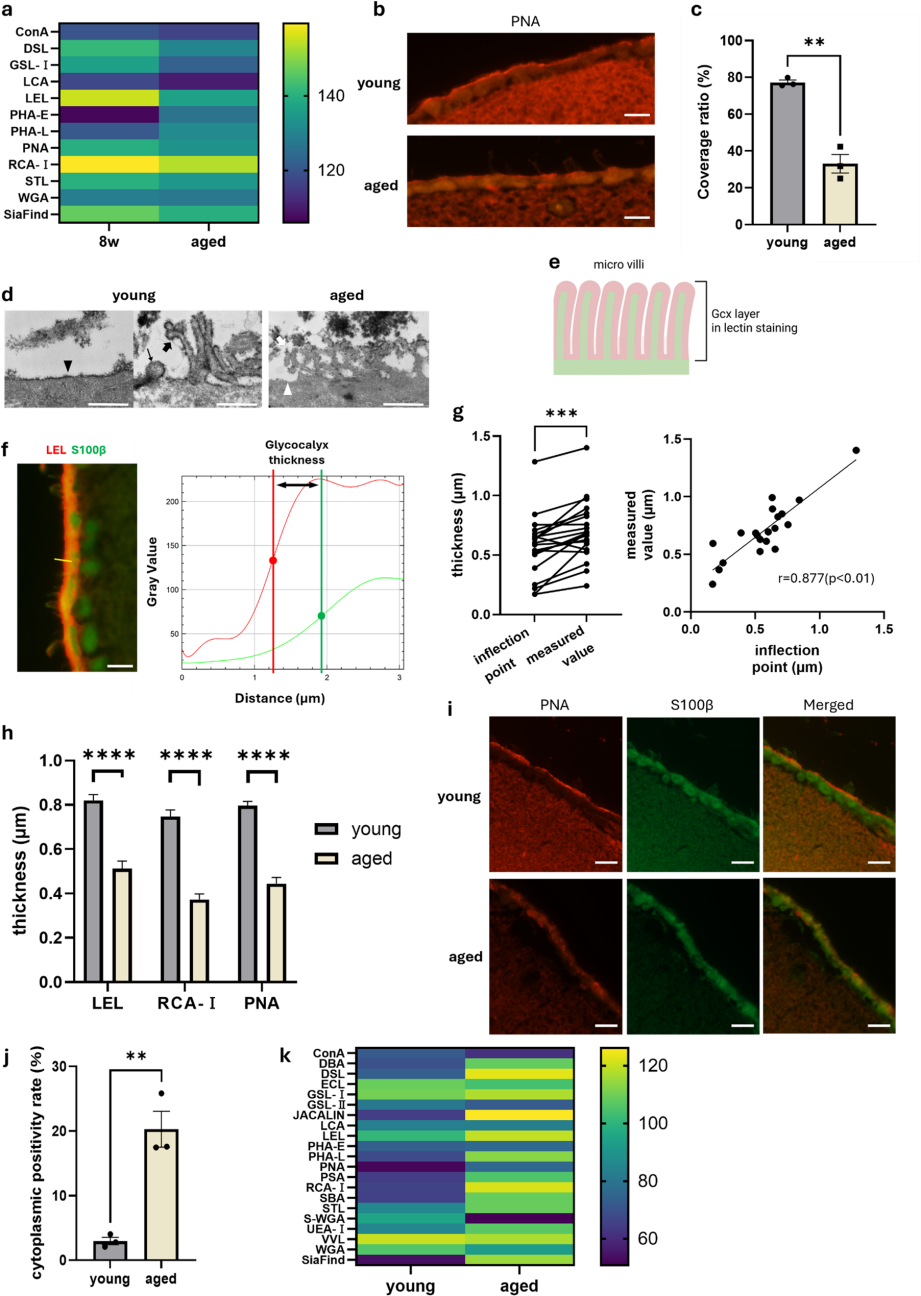
Comparison of ependymal Gcx between young adult and aged mice. (**a**) Heatmap showing the mean fluorescence intensity per pixel of the ependymal glycocalyx (Gcx) for 21 lectins in young adult and aged mice (*n* = 3 mice per group; 10 cells per mouse). (**b**) Representative PNA-based fluorescence images of the ependymal Gcx in young adult (top) and aged (bottom) mice. White scale bar, 5 μm. (**c**) Quantification of Gcx coverage along the periventricular circumference of the lateral ventricle (*n* = 3 mice per group; two-sided t-test; mean ± S.E.M.). (**d**) Transmission electron microscopy (TEM) images of ependymal cells. In young adults (left), the Gcx is visible on the apical surface (black arrowheads), microvilli (black arrows), and cilia (small black arrow). In aged mice (right), loss of the Gcx is evident on the apical surface (white arrowheads) and microvilli (white arrows). White scale bar, 500 nm. (**e**) Schematic illustration of the ependymal surface Gcx based on lectin staining and TEM. (**f**) Double fluorescence staining for LEL (red) and S100β (green) (left) with corresponding fluorescence intensity profiles (right; red = LEL, green = S100β; dots indicate inflection points). White scale bar, 5 μm. (**g**) Correlation between inflection point distance and manually measured Gcx thickness using PNA staining (*n* = 20 measurement sites; Pearson correlation). (**h**) Comparison of Gcx thickness between young adult and aged mice determined by inflection point distances for LEL, PNA, and RCA-I (*n* = 3 mice per group; 16 sites per mouse; two-sided t-test; mean ± S.E.M.). (**i**) Representative double fluorescence staining for PNA (red) and S100β (green) showing enhanced cytoplasmic PNA positivity in aged ependymal cells. White scale bar, 5 μm. (**j**) Cytoplasmic PNA-positive rate along the ventricular wall (*n* = 3 per group; two-sided t-test; mean ± S.E.M.). (**k**) Heatmap of mean fluorescence intensity in the ependymal cytoplasm for 21 lectins (*n* = 3 per group; 10 cells per mouse)

As performed in young mice, the mean fluorescence intensity of the ependymal Gcx was measured in aged mice. Enhanced intensities were observed with PHA-E and PHA-L staining; however, many lectins, including SiaFind and LEL, tended to be lower. The pattern of lectin positivity and negativity remained unchanged across groups, regardless of lectin type (Fig. 2a). Morphologically, the ependymal Gcx in aged mice appeared markedly thinned, and widespread detachment of the layer was observed (Fig. 2b). Quantitative analysis of Gcx coverage along the ventricular circumference revealed a significant decrease from 77.25 ± 1.31% in young mice to 33.06 ± 5.05% in aged mice (*p* < 0.0011) (Figure 2c). These age-related changes were confirmed via TEM imaging (Fig. 2d). In young adult mice, TEM revealed that Gcx covered not only the apical surface of the ependymal cells (black arrow) but also the microvilli (black arrow head) and cilia (small black arrow), whereas in aged mice, partial loss of Gcx was observed. Furthermore, comparison between TEM and lectin-stained images, based on differences in the thickness of the glycan layer, suggested that the Gcx layer observed in lectin staining corresponds to the layer that includes the microvilli (Fig. 2e). Using lectin-stained images, the thickness of the Gcx layer containing microvilli was evaluated by measuring the distance between inflection points in the fluorescence intensity profile (Fig. 2f). The validity of this method was confirmed using PNA staining, which were approximately 20% lower than the validity of direct measurements but displayed a strong positive correlation (*r* = 0.877, *p* < 0.01), validating this metric for relative thickness quantification and supporting its utility for relative Gcx thickness assessment (Fig. 2g). Using this method, significant thinning was observed in aged mice across all three representative lectins: LEL (0.821 ± 0.025 vs. 0.512 ± 0.034 μm), PNA (0.746 ± 0.031 vs. 0.373 ± 0.025 μm), and RCA-I (0.796 ± 0.020 vs. 0.445 ± 0.028 μm) (Figure 2h).

Consistent with this baseline pattern, aged mice exhibited ectopic cytoplasmic PNA signals, with PNA-positive cells increasing from 2.97 ± 0.57% to 20.30 ± 2.78% in young adult mice (Fig. 2i, j), indicating intracellular retention of Galβ1-3GalNAc-terminated glycans. Enhanced cytoplasmic binding was observed for many other lectins, except for S-WGA, as determined via comparative fluorescence intensity analysis (Fig. 2k).

### Dynamic changes in the ependymal Gcx following IVH

**Fig. 3:**
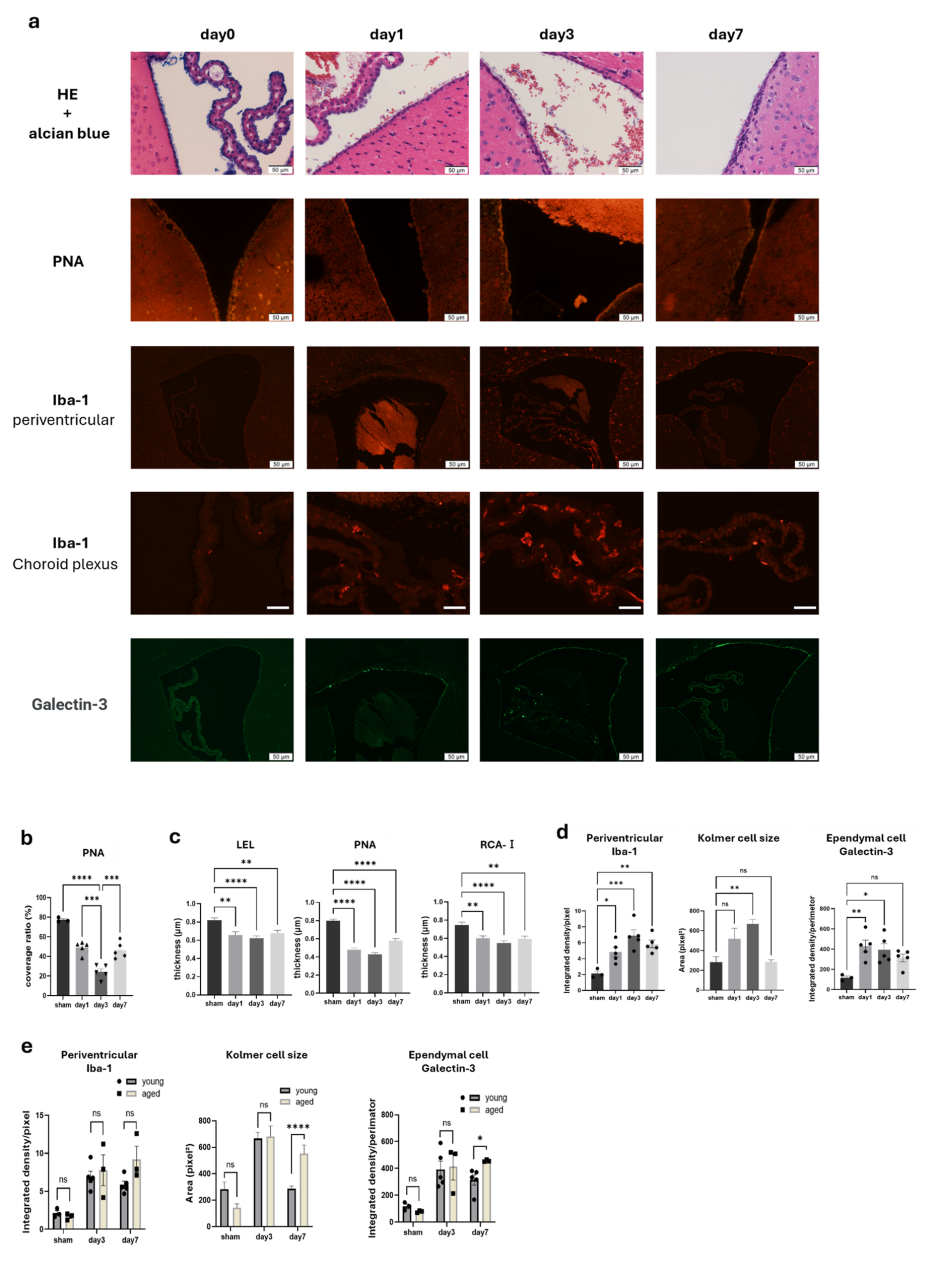
Time-course changes in ependymal Gcx and inflammation following IVH. (**a**) Representative images showing the time-course (day 0, 1, 3, 7) of ependymal Gcx (Alcian blue and PNA staining), periventricular inflammation (Iba-1, Galectin-3), and choroid plexus inflammation (Iba-1) in the IVH model. White scale bar: 10 μm. (**b**–**e**) Quantitative analyses. Unless otherwise noted, data are presented as mean ± S.E.M., analyzed using one-way ANOVA with Dunnett’s post hoc test, with *n* = 3 mice for sham and *n* = 5 mice for IVH day 1, 3, and 7 (see Statistical analysis for details). (**b**) Time-course of ependymal Gcx coverage rate during the acute phase of IVH, as assessed using PNA staining. (**c**) Time-dependent changes in ependymal Gcx thickness during the acute phase of IVH, measured using inflection-point distances with LEL, PNA, and RCA-I (16 measurement sites per mouse). (**d**) Temporal progression of inflammation during the acute phase of IVH: (left) Iba-1 fluorescence intensity per pixel in the periventricular parenchyma; (center) size of Iba-1-positive Kolmer cells in the choroid plexus (cell counts: sham *n* = 13, day 1 *n* = 12, day 3 *n* = 133, day 7 *n* = 124); (right) Galectin-3 fluorescence intensity per unit ventricular circumference in ependymal cells. (**e**) Comparison of acute inflammatory responses between young and aged mice at days 0, 3, and 7 after IVH: (left) Iba-1 fluorescence intensity per pixel in the periventricular parenchyma; (center) size of Iba-1–positive Kolmer cells (young: *n* = 13, 133, 124; aged: *n* = 7, 32, 24); (right) Galectin-3 fluorescence intensity per unit ventricular circumference in ependymal cells (young *n* = 5, aged *n* = 3)

Time-course analysis following IVH induction revealed a rapid thinning and loss of the ependymal Gcx layer, as demonstrated via Alcian blue and PNA staining (Fig. 3a). Quantification of Gcx detachment based on PNA staining showed maximal damage on day 3 (mean ± S.E.M: 24.3 ± 3.2%, vs. sham 77.3 ± 1.3%; *p* < 0.001), with partial recovery by day 7 (46.3 ± 3.7%; Fig. 3b; detailed values in Supplementary Table 1, Additional file 2). However, quantitative analysis of Gcx thickness using LEL, PNA, and RCA-I demonstrated a sustained reduction from day 1 through day 7 compared with sham controls (Fig. 3c; Supplementary Table 2, Additional file 2).

The periventricular inflammatory response—assessed via Iba-1-positive microglia and Galectin-3 expression in ependymal cells—together with choroid plexus inflammation assessed using the size of Iba-1-positive Kolmer cells, peaked at days 1–3 and showed partial attenuation by day 7 (Fig. 3d; Supplementary Table 3, Additional file 2). In contrast, in aged IVH model mice, neither periventricular Iba-1 nor ependymal Galectin-3 signals showed attenuation by day 7. Although Iba-1 intensity remained elevated, the difference from young mice was not statistically significant (5.85 ± 0.48 vs. 9.20 ± 1.71; *p* = 0.0547). In contrast, both Kolmer cell size and ependymal Galectin-3 expression were significantly higher in aged mice than in young controls at day 7 (Kolmer cell size: 283.7 ± 19.2 vs. 552.6 ± 64.4; *p* < 0.001; Galectin-3: 314.2 ± 38.5 vs. 456.7 ± 45.1; *p* = 0.0324), whereas no significant differences were observed between the two groups at days 1 or 3.

### Multilayered molecular remodeling of aged ependymal cells revealed via integrated single-cell transcriptomics

To investigate age-related transcriptomic changes in murine ependymal cells, we integrated 9,406 cells from five public single-cell RNA-seq datasets (SCP565, SRP135960, GSE74672, SCP318, and PMID_32669714_FACS). This analysis revealed pronounced alterations in the expression of gene sets related to glycan biosynthesis, glycan sialylation, desialylation, vesicular transport, and inflammatory responses in aged ependymal cells compared with their younger cells.

**Fig. 4:**
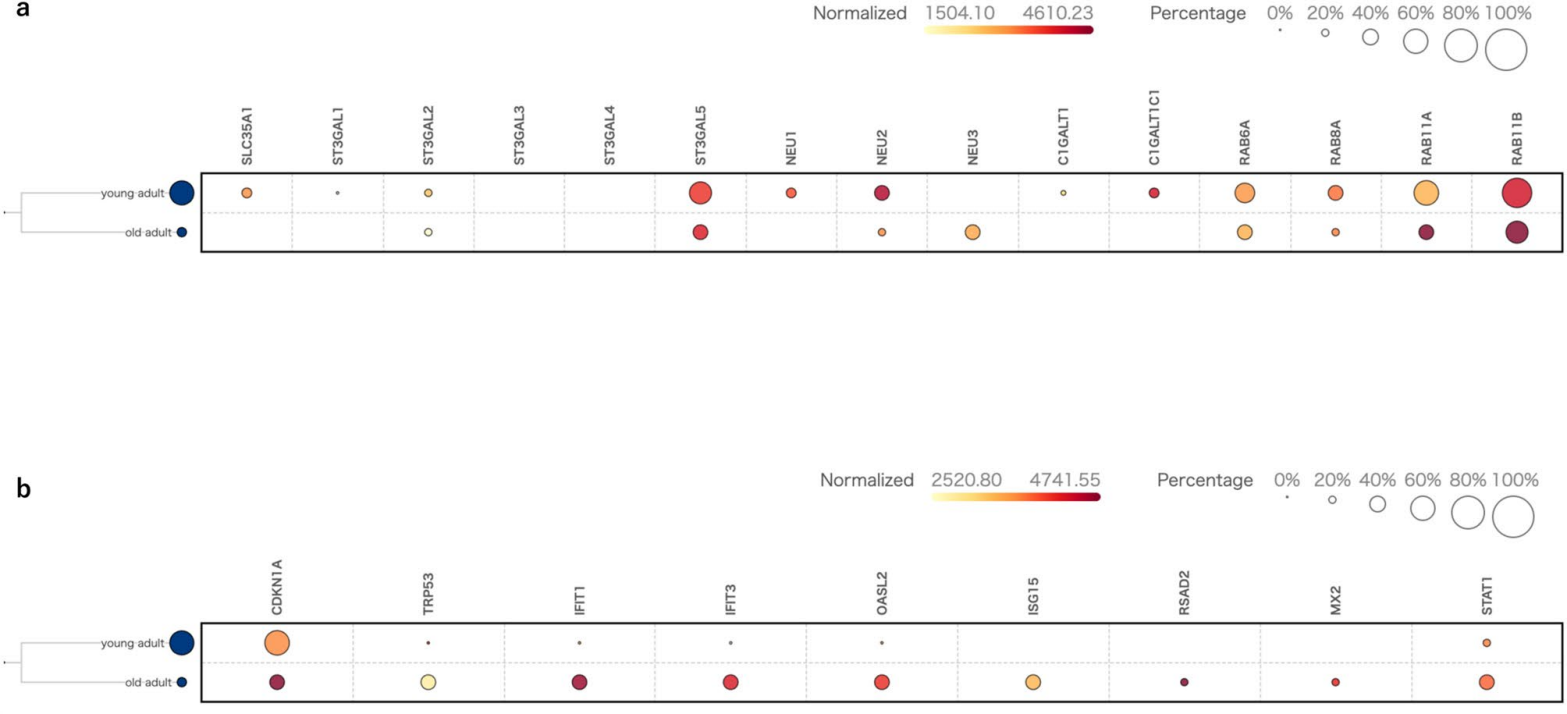
Multilayered molecular remodeling of aged ependymal cells revealed by integrated single-cell transcriptomics. Bubble heatmap depicting the proportion of cells expressing each gene (dot size) and the normalized mean expression level (color intensity) in young and aged adult mice. (**a**) Expression profiles of genes related to glycan biosynthesis, glycan sialylation, desialylation, and vesicular transport. (**b**) Expression profiles of genes associated with cellular senescence markers and interferon-stimulated inflammatory responses

Comprehensive pathway analysis showed that the CMP-sialic acid transporter SLC35A1 was decreased. Within the sialyltransferase family, the proportion of expressing cells (reflected by bubble size) and the normalized mean expression of ST3GAL1 decreased, whereas ST3GAL2/3/4 remained unchanged and ST3GAL5 increased in terms of normalized mean expression. Among sialidases, NEU1 and NEU2 decreased in both measures, whereas NEU3 increased. The core 1 O-glycan enzyme C1GALT1 and its molecular chaperone C1GALT1C1 were downregulated in both the proportion of expressing cells and normalized mean expression. For vesicular transport-related genes, RAB6A and RAB8A were reduced, whereas RAB11A and RAB11B showed a lower fraction of expressing cells but higher per-cell expression in ependymal cells when analyzed in a cell type-stratified manner (Fig. 4a).

Classical markers of cellular senescence such as Cdkn1a and Trp53, together with a broad set of interferon-stimulated genes (Ifit1, Ifit3, Oasl2, Isg15, Rsad2, Mx2, Stat1), were upregulated in aged samples (Fig. 4b).

## Discussion

**Fig. 5:**
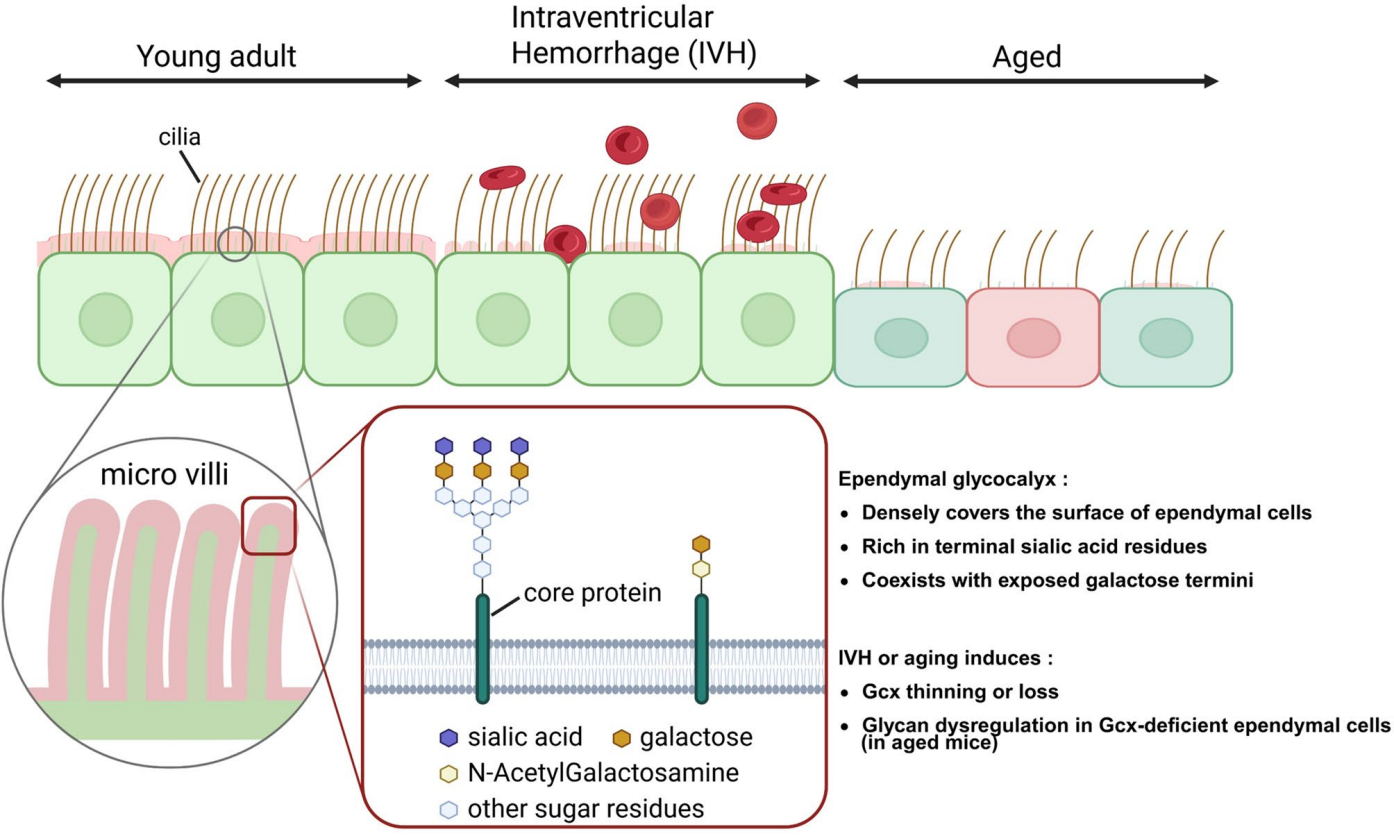
Age- and IVH-associated alterations in the ependymal glycocalyx. Schematic illustration of ependymal Gcx changes in aging and IVH. In the young state, the ependymal surface is covered by a dense Gcx rich in sialic acid and galactose residues. Aging leads to Gcx thinning and altered glycan composition, whereas IVH causes acute Gcx loss and epithelial denudation. These changes may compromise the integrity of the CSF–brain barrier and promote neuroinflammation

This study provides a comprehensive characterization of the glycan profile of the ependymal Gcx in the murine brain and demonstrates its dynamic alterations in response to aging and acute brain injury (Fig. 5).

Notably, we adapted a method originally developed for evaluating Gcx thickness via two-photon microscopy [16, 17] and applied it, for the first time, to fluorescence immunohistochemistry. This enabled the objective quantification of glycan distribution beyond conventional qualitative assessments. Kutuzov et al. [18] stated that knowledge of the glycocalyx partition coefficient may increase the accuracy of thickness measurements, as molecular accessibility to the glycocalyx is molecule-specific. In contrast, our approach focused on structural alterations defined by lectin-derived fluorescence profiles, thereby providing complementary information on Gcx remodeling. This approach supports the emerging concept of the “glycocode”—where changes in glycan composition reflect cellular state—and introduces a novel perspective in neuroglycobiology.

Previous studies [3, 6, 7], including ours, have shown that the ependymal Gcx is rich in sialylated glycans. Besides, sialic acid is a key component of vascular endothelial Gcx and plays critical roles in intercellular interactions, glycoprotein binding, and the regulation of vascular permeability [19, 20]. It is plausible that in the ependyma, sialic acid contributes similarly to selective permeability. Moreover, its hydrophilic and negatively charged nature may reduce friction with CSF, acting as a lubricant to support smooth CSF flow. Notably, electrohydrodynamic studies suggest that the negative charge of the Gcx may directly facilitate CSF perfusion [21].

Although previous studies [3, 4, 6] have indicated that the ependymal Gcx is PNA-negative unless treated with neuraminidase, our study identified readily detectable PNA-positivity without enzymatic treatment. This discrepancy may arise from differences in tissue preparation; paraffin sectioning often compromises glycan epitopes, whereas our use of cryosections better preserved Gcx structure in a near-native state [22, 23]. Meanwhile, reports of PNA-positive Gcx in vascular endothelium are extremely limited. No studies have demonstrated PNA positivity in normal endothelial Gcx without neuraminidase treatment. Where such staining has been observed, it has been confined to endothelial cells located in the central regions of certain tumors [24]. Our findings suggest that the ependymal Gcx, despite being rich in sialic acid, contains an unusually high abundance of galactose-terminated glycans lacking sialic acid capping. This glycan configuration may represent a structural adaptation that enables molecular exchange with the brain parenchyma while preserving selective barrier function, distinguishing it from vascular endothelium.

Age-related thinning and decreased coverage of the ependymal cell Gcx, accompanied by a reduction in terminal galactose residues, α2,3-linked sialic acids, and poly-N-acetyllactosamine structures, suggest impaired molecular exchange with the brain parenchyma, disruption of barrier function, and dysfunction in CSF circulation. Notably, sialic acids function as “self” markers contributing to the maintenance of immune tolerance. Therefore, the loss of this “sialic acid shield” may increase susceptibility to immune factors—including complement activation [25]—and act as a trigger or exacerbating factor for age-associated chronic neuroinflammation. Collectively, these findings suggest that the degradation of the Gcx on the ependymal surface may contribute to the disruption of homeostasis in the cerebral microenvironment.

In addition, cytoplasmic PNA binding, which was absent in young mice, was frequently observed in the ependymal cells of aged mice. In most of these PNA-positive cells, thinning or partial loss of the apical Gcx was detected. Furthermore, several lectins—including those specific for α2,3-linked sialic acids—exhibited markedly enhanced intracellular staining intensity in aged ependymal cells, indicating glycan-related remodeling within the ependymal cells.

Transcriptomic analysis showed that, compared with young cells, aged murine ependymal cells displayed changes in gene sets related to glycan biosynthesis, glycan sialylation, desialylation, vesicular transport, and inflammatory responses. Downregulation of the sialylation genes ST3GAL1 and SLC35A1, together with reduced expression of C1GALT1 and C1GALT1C1 (key enzymes for Core-1 O-glycan formation), indicates diminished capacity for glycan synthesis. Altered expression of Rab GTPases—including decreased RAB6A/8A and per-cell upregulation of RAB11A/B revealed by cell type-stratified analysis—points to age-related reorganization of membrane trafficking. In contrast, decreased NEU1 and NEU2, which localize mainly to lysosomes and the cytosol, alongside increased NEU3, a plasma-membrane sialidase [26], suggest a relative enhancement of membrane desialylation. These findings lend support to the concept of disrupted glycoprotein homeostasis in aged ependymal cells [27]. Moreover, upregulation of Cdkn1a, Trp53, and multiple interferon-stimulated genes accords with the concept of IFN-aging, an inflammation-linked process of aging [28].

Taken together, these observations suggest that ependymal cells undergo multilayered, bidirectional molecular remodeling with aging, involving glycan biosynthesis, vesicular trafficking, sialic-acid metabolism, and innate immune activation. Consistent with glycosylation changes reported in aging and neurodegeneration [29], thinning or loss of the ependymal Gcx may compromise ependymal cell integrity, potentially disrupting intercellular adhesion and signaling required to maintain the subventricular zone neural stem-cell niche and impairing ciliary function [30, 31]. These alterations may, in turn, contribute to disturbances of brain microenvironmental homeostasis and underlie age-associated brain pathologies, including ventricular enlargement and impaired metabolic waste clearance, characteristic of conditions such as normal-pressure hydrocephalus and Alzheimer’s disease.

Our IVH model revealed acute-phase disruption of the ependymal Gcx. Decreased binding of LEL, PNA, and RCA-I following IVH suggests Gcx damage and subsequent barrier failure due to hemorrhagic insult, with recovery requiring an extended time. In agreement with prior studies [32], our data showed that IVH-induced inflammation was prolonged in aged mice. These results suggest the possibility that age-related Gcx loss and thinning not only impedes recovery from acute brain injury but also contributes to persistent inflammation and deterioration of CSF dynamics [33]. Previous studies have shown that IVH can damage ependymal cells and compromise their integrity [34, 35]. In our study, however, paraffin sections used for parallel morphological evaluation showed only minimal denudation (≤ 1.6% of the total ventricular surface circumference) in days 1, 3, and 7 in aged mice. This discrepancy from previous studies may be partly explained by differences in the experimental subjects, as those studies examined neonatal brains, whereas the present study used adult mice, whose ependymal layer is structurally more mature and resistant to mechanical or inflammatory injury. In contrast, frozen sections used for lectin staining displayed higher apparent denudation (approximately 2–11%), which we attribute to freezing or sectioning artifacts rather than disease-related ependymal loss. Because regions of cell loss were explicitly excluded from Gcx coverage measurements, these data indicate that the observed thinning and detachment of the Gcx occur mainly on structurally intact ependyma. Taken together, these findings suggest that hemolysate-induced inflammation and junctional stress initially induce thinning and loss of the ependymal Gcx, with overt ependymal loss representing a later or more severe stage of injury. This concept is further supported by experimental evidence showing that intracerebroventricular administration of neuraminidase removes sialic acids and subsequently causes ependymal cell detachment [25], indicating that glycocalyx damage—particularly sialic acid loss—can precede and promote ependymal denudation. The novelty of this study lies in the comprehensive and quantitative profiling of both apical and intracellular glycans of the ependymal Gcx using cryosections. Furthermore, it uniquely demonstrates that these glycan structures undergo dynamic changes in response to aging and brain injury. Maintaining or restoring the integrity of the ependymal Gcx may contribute to the preservation and regeneration of ependymal cell function. In addition, this study provides a foundational framework for the comprehensive understanding of the roles of ependymal glycans in various processes, including CSF circulation, cell adhesion, and neuroimmune regulation, spanning from molecular mechanisms to systems-level neurobiology. Furthermore, these findings suggest the potential for developing novel anti-aging strategies and therapeutic approaches targeting glycan modifications in neurodegenerative diseases.

### Limitations

This study was limited to histochemical analysis using lectins and electron microscopy in murine models, and caution is required when extrapolating to human physiology. In addition, owing to potential overlap in lectin-binding specificities, detailed structural identification of individual glycans may require complementary high-resolution glycomic approaches such as mass spectrometry. The metric used in this study—the distance between fluorescence intensity inflection points—reflects relative Gcx thickness including the microvilli but does not represent absolute physical dimensions. Moreover, we identified ependymal cells based on S100β immunoreactivity. Although S100β is widely used as a marker of mature ependymal cells, potential changes in its subcellular distribution or polarity under different conditions cannot be excluded, and such alterations may affect the accuracy of thickness measurements. In addition, we did not use anticoagulants to reproduce the natural composition of IVH and avoid potential anti-inflammatory or ion-modifying effects of heparin and other anticoagulants. Blood was injected within seconds of collection to minimize clot formation. Although we cannot entirely exclude contributions of micro-clotting or hemolysis to the inflammatory response, all injections were administered under the same standardized conditions, and any such effects are expected to be minimized and comparable among animals. Moving forward, several approaches could enhance the interpretability and applicability of these findings. Integration of structural and functional analyses—such as tracer-based CSF clearance assays, permeability measurements, and direct assessment of ciliary function—will help delineate the physiological consequences of Gcx alterations. Causal investigations using gain- or loss-of-function strategies, conditional gene manipulation, or targeted enzymatic degradation can further clarify how Gcx remodeling affects disease severity, recovery, or therapeutic response. Pharmacological modulation with agents such as rapamycin or glycosylation-targeted compounds may inform strategies to preserve or restore Gcx integrity. Finally, combining glycomic analyses with single-cell or spatial transcriptomics may uncover cell-type-specific regulation of glycan biosynthesis and its link to neuroimmune and barrier functions.

## Conclusions

This study demonstrated that the ependymal Gcx in the murine brain exhibits a glycan profile that likely contributes to molecular exchange with the brain parenchyma, CSF circulation, and the maintenance of barrier integrity under physiological conditions. In addition, we captured the dynamic alterations in the ependymal glycan profile associated with aging and acute brain injury. Notably, the preservation of ependymal cell function in the aging brain and its restoration following brain injury may represent critical therapeutic targets for delaying the progression of neurodegenerative processes. By elucidating the previously underexplored role of ependymal glycans in maintaining the homeostasis of the brain microenvironment, this study establishes a foundation for a new research direction at the intersection of neuroscience and neuropathology.

## Supplementary Information

Below is the link to the electronic supplementary material.

**Supplementary Material 1:** Workflow for measurement of ependymal glycocalyx thickness based on inflection-point distance in ImageJ. (a) Stepwise procedure for evaluating ependymal glycocalyx (Gcx) thickness by measuring the distance between fluorescence-intensity inflection points in ImageJ. (b) Merged image of ependymal Gcx (LEL, red) and ependymal cells (S100β, green). White scale bar: 5 μm. c, d) Plot profiles of LEL and S100β fluorescence intensities were obtained along the yellow line in (b), and 8th-degree polynomial curve fitting was performed (LEL, red; S100β, green). e) The fitted curves were overlaid. f) Inflection points were identified on the fitted curves, and the distance between the two inflection points was measured as the glycocalyx thickness. g) To validate the measurements obtained through the above step, raw data from the plot profiles were imported into GraphPad Prism and subjected to sigmoidal fitting. Inflection points were calculated by the software (e.g., lectin inflection x = 1.24 μm by 8th-degree fit vs. 1.20 μm by sigmoidal fit; S100β inflection x = 1.89 μm vs. 1.90 μm). Twenty representative curves were analyzed. Paired t-test showed no significant difference between the two methods (mean difference = 0.027 μm; 95% CI = − 0.068 to 0.122 μm; t = 0.60, df = 19; *p* = 0.56). Bland–Altman analysis demonstrated excellent agreement (bias = − 0.027 μm; SD = 0.203 μm; 95% limits of agreement = − 0.425 to + 0.371 μm). These results confirm that the ImageJ 8th-degree polynomial fit provides measurements equivalent to those obtained by sigmoidal fitting.

**Supplementary Material 2:** Supplementary table 1. Summary of ependymal Gcx coverage (%). Values are mean ± standard error of the mean (S.E.M); n = number of animals. Supplementary table 2. Ependymal Gcx thickness (µm) measured by inflection point distance. Values are mean ± S.E.M; n = number of measurement sites (16 sites per mouse; sham *n* = 48, IVH groups *n* = 80). Supplementary table 3. Inflammatory parameters after IVH. Values are mean ± S.E.M; n = number of animals for Iba-1 and Galectin-3, and number of cells for Kolmer cell size.

## Data Availability

Data is available upon reasonable request.

## Abbreviations

Gcx: Glycocalyx
CSF: Cerebrospinal fluid
IVH: Intraventricular hemorrhage
scRNA-seq: Single-cell RNA sequencing
SEM: Scanning electron microscopy
LVSEM: Low-vacuum scanning electron microscopy
S.E.M.: Standard error of the mean
RCA-Ⅰ: Ricinus communis agglutinin I
SiaFind: α2,3-linked sialic acid-specific lectins
STL: Solanum tuberosum lectin
DSL: Datura stramonium lectin (DSL)
LEL: Lycopersicon esculentum lectin
