# Supplementary figures and images for: Age‑dependent and post‑intraventricular hemorrhage remodeling of the ependymal glycocalyx in mice
Source: Fluids Barriers CNS. 2025 Nov 7;22:115. doi: 10.1186/s12987-025-00725-x (PMC12595828; doi:10.1186/s12987-025-00725-x)

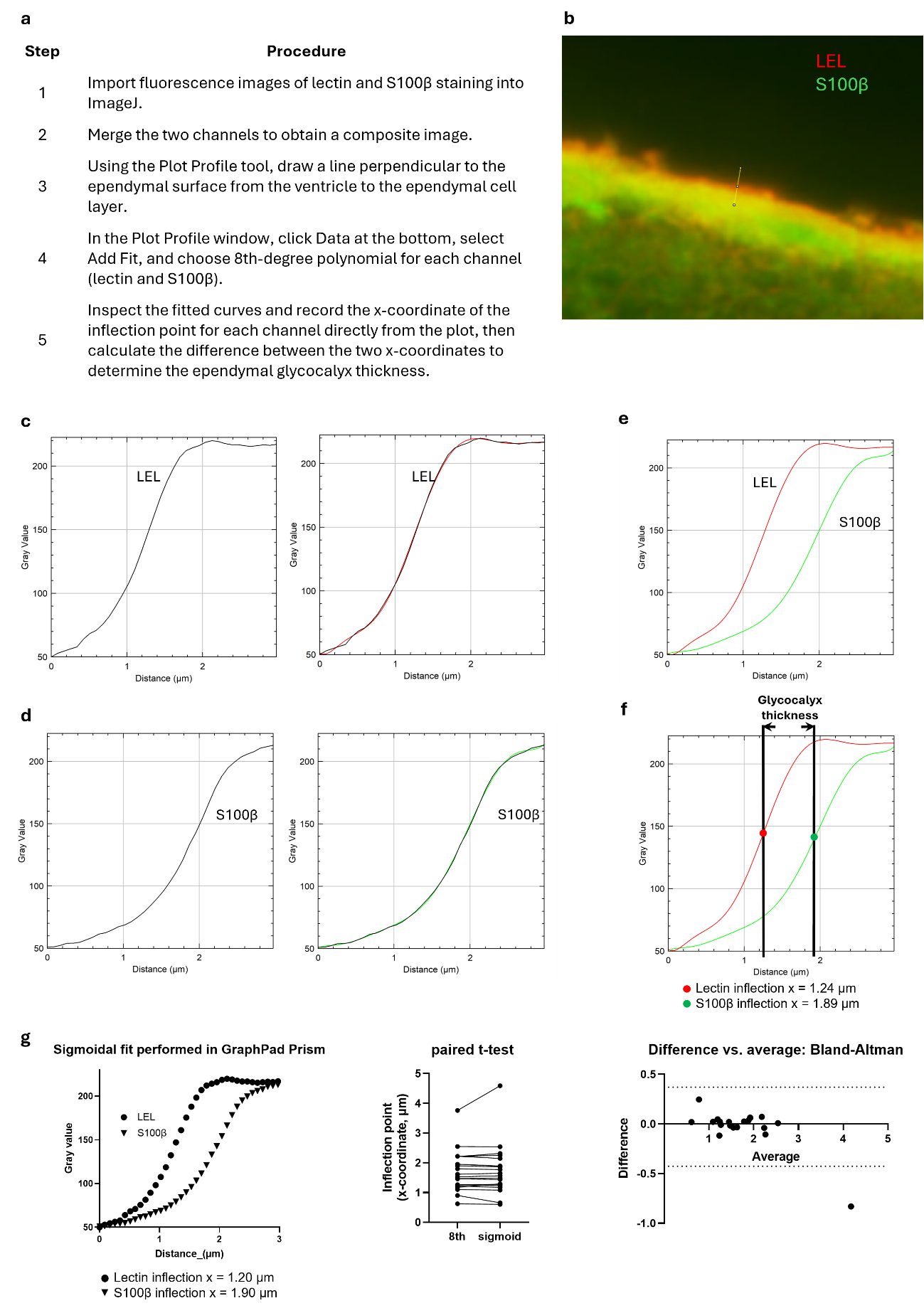

Supplement: Supplementary file 1 — Supplementary Material 1: Workflow for measurement of ependymal glycocalyx thickness based on inflection-point distance in ImageJ. (a) Stepwise procedure for evaluating ependymal glycocalyx (Gcx) thickness by measuring the distance between fluorescence-intensity inflection points in ImageJ. (b) Merged image of ependymal Gcx (LEL, red) and ependymal cells (S100β, green). White scale bar: 5 μm. c, d) Plot profiles of LEL and S100β fluorescence intensities were obtained along the yellow line in (b), and 8th-degree polynomial curve fitting was performed (LEL, red; S100β, green). e) The fitted curves were overlaid. f) Inflection points were identified on the fitted curves, and the distance between the two inflection points was measured as the glycocalyx thickness. g) To validate the measurements obtained through the above step, raw data from the plot profiles were imported into GraphPad Prism and subjected to sigmoidal fitting. Inflection points were calculated by the software (e.g., lectin inflection x = 1.24 μm by 8th-degree fit vs. 1.20 μm by sigmoidal fit; S100β inflection x = 1.89 μm vs. 1.90 μm). Twenty representative curves were analyzed. Paired t-test showed no significant difference between the two methods (mean difference = 0.027 μm; 95% CI = − 0.068 to 0.122 μm; t = 0.60, df = 19; p = 0.56). Bland–Altman analysis demonstrated excellent agreement (bias = − 0.027 μm; SD = 0.203 μm; 95% limits of agreement = − 0.425 to + 0.371 μm). These results confirm that the ImageJ 8th-degree polynomial fit provides measurements equivalent to those obtained by sigmoidal fitting. [file 12987_2025_725_MOESM1_ESM.docx]
