# Supplementary material for: Age‑dependent and post‑intraventricular hemorrhage remodeling of the ependymal glycocalyx in mice
Source: Fluids Barriers CNS. 2025 Nov 7;22:115. doi: 10.1186/s12987-025-00725-x (PMC12595828; doi:10.1186/s12987-025-00725-x)
Supplement: Supplementary file 2 — Supplementary Material 2: Supplementary table 1. Summary of ependymal Gcx coverage (%). Values are mean ± standard error of the mean (S.E.M); n = number of animals. Supplementary table 2. Ependymal Gcx thickness (µm) measured by inflection point distance. Values are mean ± S.E.M; n = number of measurement sites (16 sites per mouse; sham n = 48, IVH groups n = 80). Supplementary table 3. Inflammatory parameters after IVH. Values are mean ± S.E.M; n = number of animals for Iba-1 and Galectin-3, and number of cells for Kolmer cell size. [file 12987_2025_725_MOESM2_ESM.docx]

**Supplementary Table 1**

| Group | Mean ± SEM | n |
| --- | --- | --- |
| Sham | 77.26 ± 1.31 | 3 |
| Day 1 | 48.97 ± 2.82 | 5 |
| Day 3 | 24.26 ± 3.16 | 5 |
| Day 7 | 46.28 ± 3.73 | 5 |

*Values are mean ± standard error of the mean (S.E.M); n = number of animals.*

**Supplementary Table 2**

| Lectin | Sham | Day 1 | Day 3 | Day 7 |
| --- | --- | --- | --- | --- |
| LEL | 0.821 ± 0.026 | 0.659 ± 0.032 | 0.622 ± 0.027 | 0.678 ± 0.032 |
| PNA | 0.796 ± 0.020 | 0.477 ± 0.023 | 0.428 ± 0.020 | 0.578 ± 0.023 |
| RCA-I | 0.747 ± 0.030 | 0.602 ± 0.024 | 0.548 ± 0.028 | 0.595 ± 0.029 |

*Values are mean ± S.E.M; n = number of measurement sites (16 sites per mouse; sham n = 48, IVH groups n = 80).*

**Supplementary Table 3**

| Parameter | Group | Day | Mean ± SEM | n |
| --- | --- | --- | --- | --- |
| Iba-1 intensity (integrated density/pixel) | Young | Sham | 2.76 ± 0.33 | 3 |
|  |  | Day 3 | 6.89 ± 0.73 | 3 |
|  |  | Day 7 | 5.85 ± 0.48 | 3 |
|  | Aged | Sham | 1.67 ± 0.24 | 3 |
|  |  | Day 3 | 7.76 ± 2.03 | 3 |
|  |  | Day 7 | 9.20 ± 1.71 | 3 |
| Kolmer cell size (pixel²) | Young | Sham | 282.1 ± 55.5 | 13 |
|  |  | Day 3 | 666.4 ± 46.3 | 133 |
|  |  | Day 7 | 287.3 ± 19.2 | 124 |
|  | Aged | Sham | 144.3 ± 28.5 | 7 |
|  |  | Day 3 | 681.2 ± 79.8 | 32 |
|  |  | Day 7 | 552.6 ± 64.4 | 24 |
| Galectin-3 intensity (integrated/perimeter) | Young | Sham | 117.7 ± 17.1 | 3 |
|  |  | Day 3 | 393.1 ± 61.2 | 5 |
|  |  | Day 7 | 314.2 ± 38.5 | 5 |
|  | Aged | Sham | 78.3 ± 4.43 | 3 |
|  |  | Day 3 | 414.1 ± 100.4 | 3 |
|  |  | Day 7 | 456.7 ± 45.1 | 3 |

*Values are mean ± S.E.M; n = number of animals for Iba-1 and Galectin-3, and number of cells for Kolmer cell size.*
